# Adipokine chemerin overexpression in trophoblasts leads to dyslipidemia in pregnant mice: implications for preeclampsia

**DOI:** 10.1186/s12944-023-01777-4

**Published:** 2023-01-25

**Authors:** Lunbo Tan, Zijun Ouyang, Zhilong Chen, Fen Sun, Haichun Guo, Feng Wang, Monique Mulder, Yuan Sun, Xifeng Lu, Jian V. Zhang, A. H. Jan Danser, Koen Verdonk, Xiujun Fan, Qing Yang

**Affiliations:** 1 College of Veterinary Medicine, Hunan Agricultural University, Changsha, 410128 China; 2 Center for Energy Metabolism and Reproduction, Institute of Biomedicine and Biotechnology, Shenzhen Institute of Advanced Technology, Chinese Academy of Sciences, Shenzhen, 518055 China; 3 Division of Vascular Medicine and Pharmacology, Department of Internal Medicine, Erasmus MC, Rotterdam, Netherlands; 4 School of Food and Drug, Shenzhen Polytechnic, Institute of Marine Biomedicine, Shenzhen, 518055 China; 5 Changsha Hospital for Maternal and Child Health Care, Changsha, 410007 China; 6 Department of Obstetrics and Gynecology, Shenzhen Hengsheng Hospital, Shenzhen, 518115 China; 7 Clinical Research Center, The First Affiliated Hospital of Shantou University Medical College, Shantou, 515041 China

**Keywords:** Chemerin, Preeclampsia, Dyslipidemia, Placenta, Trophoblast, Phospholipids

## Abstract

**Background:**

The adipokine chemerin regulates adipogenesis and the metabolic function of both adipocytes and liver. Chemerin is elevated in preeclamptic women, and overexpression of chemerin in placental trophoblasts induces preeclampsia-like symptoms in mice. Preeclampsia is known to be accompanied by dyslipidemia, albeit via unknown mechanisms. Here, we hypothesized that chemerin might be a contributor to dyslipidemia.

**Methods:**

Serum lipid fractions as well as lipid-related genes and proteins were determined in pregnant mice with chemerin overexpression in placental trophoblasts and chemerin-overexpressing human trophoblasts. In addition, a phospholipidomics analysis was performed in chemerin-overexpressing trophoblasts.

**Results:**

Overexpression of chemerin in trophoblasts increased the circulating and placental levels of cholesterol rather than triglycerides. It also increased the serum levels of lysophosphatidic acid, high-density lipoprotein cholesterol (HDL-C), and and low-density lipoprotein cholesterol (LDL-C), and induced placental lipid accumulation. Mechanistically, chemerin upregulated the levels of peroxisome proliferator-activated receptor g, fatty acid-binding protein 4, adiponectin, sterol regulatory element-binding protein 1 and 2, and the ratio of phosphorylated extracellular signal-regulated protein kinase (ERK)1/2 / total ERK1/2 in the placenta of mice and human trophoblasts. Furthermore, chemerin overexpression in human trophoblasts increased the production of lysophospholipids and phospholipids, particularly lysophosphatidylethanolamine.

**Conclusions:**

Overexpression of placental chemerin production disrupts trophoblast lipid metabolism, thereby potentially contributing to dyslipidemia in preeclampsia.

**Supplementary Information:**

The online version contains supplementary material available at 10.1186/s12944-023-01777-4.

## Background

Preeclampsia is a progressive, multi-systemic disorder which causes a high prevalence of pregnancy-related morbidity and mortality [1]. Clinically, preeclampsia is defined as new onset of hypertension after 20 weeks of gestation and either proteinuria or any of new onset hemolysis, hepatic impairment, renal dysfunction, headache, pulmonary congestion or low platelets [2, 3]. The exact pathophysiology is still unknown. Increasing evidence reveals an association between this pathological condition and an imbalance in lipid regulation. Previous studies revealed that preeclamptic patients have a higher serum level of triglycerides, total cholesterol, phospholipids and low-density lipoprotein cholesterol (LDL-C) [4–9].

Chemerin, a small chemotactic adipokine, affects blood pressure, cholesterol levels, adipose tissue function, and insulin sensitivity [10]. It acts through three receptors, C-C motif chemokine receptor-like 2 (CCRL2), chemerin receptor 1, also named chemerin chemokine-like receptor 1 (CMKLR1), and chemerin receptor 2, also named G protein-coupled receptor 1 (GPR1) [11]. Blood chemerin levels are elevated in preeclampsia patients and correlate positively with preeclampsia severity We have developed a mouse model for preeclampsia by inducing chemerin overexpression in placental trophoblasts. These anmials developed hypertension and proteinuria, but they also showed severe placental vascular damage. Significant embryonic growth restriction and lethality were observed in this animal model [12].

We hypothesized that a high level of chemerin released from placental trophoblasts might be a risk factor contributing to dyslipidemia during preeclamptic conditions. In the current study, we therefore investigated lipid accumulation in the placenta of these mice. In addition, we performed a phospholipidomics analysis in chemerin-overexpressing human trophoblast cells.

## Materials and methods

### Production of lentiviral vectors

The construction of lentiviral vectors and the full-length chemerin overexpression model have been described in our previous work [12]. In brief, LV-mChemerin-GFP and LV-hChemerin-GFP were generated by cloning mouse chemerin or human chemerin into the lenti-viral vector respectively (LV-GFP, System Biosciences, USA). The method for producing lentiviral particles was previously described [12–14]. The titer of lentivirus was measured by using a commercial kit (Cell Biolabs Inc., San Diego, CA, USA).

### Preparation of preeclampsia mice model

Animal experiments were conducted at the Shenzhen Institutes of Advanced Technology in compliance with the Chinese Academy of Sciences board approval. CD-1 mice at the age of 8–10 weeks were purchased from Beijing Vital River Laboratory Animal Technology Co., Ltd. (Beijing, China). The procedures for generating the preeclampsia mouse model have been described previously [12–14]. The vaginal plug day was marked as the first day of pregnancy or pseudopregnancy (gestational day 1; GD1). On GD4, zona-free blastocysts were transferred to GD3 pseudopregnant mice after a 6 h-incubation with LV-mouseChemerin-GFP (Chemerin) or LV-GFP (Control). Placentas and blood samples were collected. In mouse pregnancy, the placenta grows to a complete structure and is well-maintained after GD14. It is usually prepared for delivery after GD18 [15]. Based on our previous study [12], we chose GD15 and GD18 for the following analysis. On GD15 and GD18, mice were anesthetized with 5% isoflurane administration and then euthanized by cervical dislocation. Placentas were collected for histopathological diagnosis as well as mRNA and protein analysis. Serum samples were collected for biochemical tests.

### Biochemical measurements

On GD18, blood was sampled from the mouse tail for fasting blood glucose measurement by a glucose meter (Roche, Basel, Switzerland). The serum lipid levels (including triglycerides, high-density lipoprotein cholesterol (HDL-C), cholesterol, and LDL-C) were determined using an automatic biochemical analyzer (Roche, Basel, Switzerland). Placental triglycerides and cholesterol were extracted by Folch’s method [16]. ELISA kits were utilized to detect serum levels of lysophosphatidic acid (LPA, BLUEFBIO, Shanghai, China) and chemerin (R&D Systems, Minneapolis, MN, USA), respectively.

### Histology

Mouse placentas were incubated in 4% paraformaldehyde (PFA) for 24 hours at 4 °C, followed by embedding with paraffin or O.C.T. solution (Sakura, Torrance, CA, USA).

*Oil Red O staining.* Frozen sections of the placenta, 10 μm thick, were fixed in 4% PFA and dehydrated in a gradient of sucrose. Sections were stained in Oil Red O staining solution according to the manufacturer’s instructions (Sigma-Aldrich) [17].

*Hematoxylin & eosin (H&E) staining.* Five μm thick paraffin slices were obtained. Slides were deparaffinized, and rehydrated, then stained by H&E.

*Phospholipids staining.* After fixation and dehydration, placental frozen sections were stained by paraphenylenediamine (PPD) staining method as described previously [18]. Briefly, 1% PPD (Aladdin, Shanghai, China) was added, and the slides were incubated for 10 minutes at 20 °C, then rinsed in 100% ethanol for 5 min. Sections were dried and covered with mounting solution.

The images of staining were captured by an Olympus BX53 microscope (Olympus, Japan).

### Preparation of a chemerin-overexpressing trophoblast cell model

HTR-8/SVneo cells were kept in DMEM/F12 medium with 10% fetal bovine serum (Hyclone, Logan, UT, USA) at 37 °C with 5% CO_2_. The cells were a kind gift of Dr. Charles H. Graham at Queen’s University, Kingston, Canada. Preparation of the human chemerin-overexpressing cells has been described previously [12]. The cells were infected for 72 hours, then 5 μg/mL puromycin with fresh DMEM/F12 medium with 10% fetal bovine serum for cell selection (chemerin-overexpressing cells and control cells). Stable overexpressing cells were collected and stored in liquid nitrogen for further in vitro studies. For cell experiments, the cells were seeded in 6 well plates, and cultured in DMEM/F12 medium with 10% fetal bovine serum for 48 hours. Lipid accumulation was measured in the cells by Oil Red O staining.

### Gene expression analysis

First, RNA was extracted from placentas (half of each placenta) of the control and chemerin groups and trophoblast cells (one well for each group in 6 well plates) using TRIzol® reagent (Invitrogen, Carlsbad, CA). Then, a commercial kit (Invitrogen) was used to reverse transcribe RNA (1 μg of total RNA for each cDNA reaction) to DNA templates. Finally, real-time PCR was performed using an SYBR green-based qPCR kit (TOYOBO, Osaka, Japan) and run on the LightCycler® 480 System (Roche, Pleasanton, CA, USA). Sequences of the specific primers are displayed in the Additional file 1 Supplementary Table 1. The comparative C(t) method was used to determine the mRNA levels of genes. Normalization occurred by using β-actin as an internal reference gene.

### Western blot

Total protein from both the placental samples and trophoblast cells was extracted using RIPA lysis buffer, and quantified using the Bradford method (Thermo Fisher Scientific, Waltham, MA, USA). A total of 20 μg protein of each sample was electroblotted onto PVDF membranes (Millipore, Burlington, MA) after sodium dodecyl-sulfate polyacrylamide gel electrophoresis (SDS-PAGE) separation [12]. Protein signals were developed using an enhanced chemiluminescence kit (Bio-Rad, Irvine, CA) and quantified with a ChemiDoc system (Bio-Rad, Irvine, CA). The primary antibodies and dilutions are listed as following: rabbit polyclonal antibodies against adiponectin (1:500, Proteintech, Wuhan, China), fatty acid binding protein 4 (FABP4) (1:1000, Abcam, Cambridge, UK), peroxisome proliferator activated receptor gamma (PPARg) (1:1000, Cell Signaling Technology, Boston, MA, USA), acetyl-CoA carboxylase (ACC) (1:1000, Cell Signaling), ERK1/2 (1:1000, Cell Signaling), phospho-ERK1/2 (1:1000, Cell Signaling), low density lipoprotein receptor (LDLR) (1:1000, Thermo Fisher), mouse monoclonal antibodies against sterol regulatory element-binding protein 2 (SREBP2) and sterol regulatory element-binding protein 1 (SREBP1) (1:1000, Santa Cruz Biotechnology, Santa Cruz, CA, USA), β-actin (1:5000, Sigma-Aldrich) and sortilin 1 (SORT1) (1:1000, BD Bioscience, Ann Arbor, MI, USA).

### Phospholipidomics analysis in a chemerin-overexpressing cell model

*Lipid extraction.* Intracellular lipids were extracted by the methyl-tert-butyl ether (MTBE) method [19, 20]. The chemerin-overexpressing and control trophoblast cells (3 pooled batches of cells per sample) were first resuspended in 1.5 mL methanol. Then MTBE (5 mL) was added, and the mixtures were vortexed for 1 h at 20 °C and cultured in water (1.25 mL) for 10 min. After centrifugation, the upper organic lipid phases were collected and dried in a vacuum centrifuge. Then, lipids were dissolved in CHCl_3_/methanol/water (200 μL, 60:30:4.5 by volumn) followed by phospholipidomics analysis.

*Phospholipids metabolomic analysis.* 150 μL of each lipid fraction was a mixture with a 1650 μL of chloroform/methanol/ammonium acetate (300:665:35 by volumn). Phospholipid classes were identified by electrospray ionisation tandem mass spectrometry, and quantified via using the internal standards [21], including 6.6 nmol di-14:0-phosphatidylcholine (PC), 6.6 nmol 13:0- lysophosphatidylcholine (LPC), 6.6 nmol 19:0-LPC, 3.6 nmol di-14:0- phosphatidylethanolamine (PE), 3.6 nmol 14:0-lysophosphatidylethanolamine (LPE), 3.6 nmol 18:0-LPE, 3.6 nmol di-14:0- phosphatidylglycerol (PG), 3.6 nmol 14:0- lysophosphatidylglycerol (LPG), 3.6 nmol 18:0-LPG, 3.6 nmol di14:0- phosphatidic acid (PA), 2.4 nmol di14:0- phosphatidylserine (PS), and 1.63 nmol di18:0- phosphatidylinositol (PI). The Shimadzu UFLC LC/MS system (Triple TOFTM 5600 plus; AB Sciex, Foster City, CA) combined with a C18 column (Kinetex 2.6u C18 100A 150 × 2.1 mm00F-4462-AN) was used for the analysis and identification of the lipid components. The mass spectrometry conditions were: ion source temperature, 300 °C; electrospray voltage, + 5.5 kV or − 4.5 kV; positive ion mode, collision voltage (CE), PE and LPE, + 28 V; PC and LPC, + 40 V; negative ion mode, collision voltage (CE), PI, − 58 V; PG, LPG and PA, − 57 V; PS, − 34 V. The data were analyzed qualitatively using Peakview, a companion software provided by AB Sciex, and then quantitatively using MultiQuant software.

### Statistical analysis

The data represent as mean ± SD. GraphPad Prism was used for statistical analysis (version 8, La Jolla, CA, USA). The data normality was evaluated by the Shapiro-Wilk test. Statistic difference between groups was analyzed by using the Student’s t-test, indicating as *P < 0.05 and **P < 0.01. Pearson’s correlation coefficient was calculated to assess the correlation between two variables.

## Results

### Overexpression of chemerin in trophoblasts increases lipid levels in pregnant mice

The placental specific overexpression of chemerin was first verified in trophoblasts. As shown in Additional file 1 Supplementary Fig. 1, GFP signals were observed in blastocyst trophectoderm and placentas of the control or chemerin group mice on GD18 (Additional file 1 Supplementary Fig. 1A). No GFP signals were detected in the fetus (Additional file 1 Supplementary Fig. 1B). The expression of chemerin (mRNA and protein) was increased in the placentas of the chemerin group (Additional file 1 Supplementary Fig. 1C and D). Furthermore, the circulating chemerin level was higher in the chemerin group than in the control group (Additional file 1 Supplementary Fig. 1E).

**Fig. 1:**
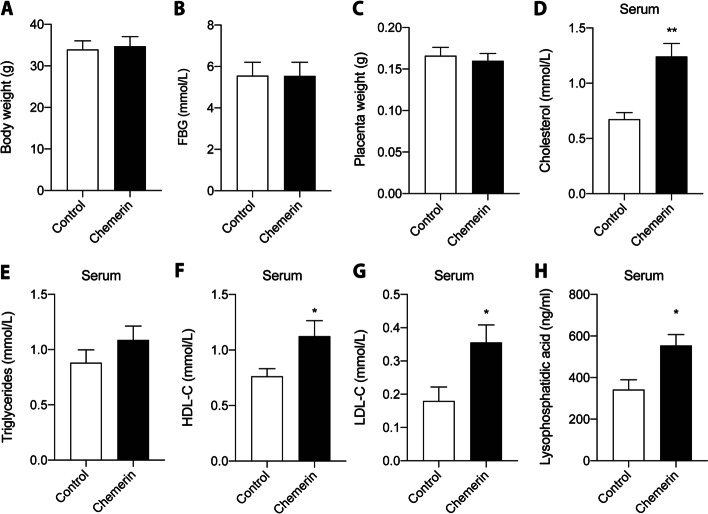
Effect of overexpression of trophoblast-specific chemerin on mouse maternal lipid levels. Placentas and blood samples were collected from preeclampsia mice or controls on GD18. **A** Body weight of the pregnant mice. **B** Fasting glucose level. **C** Mouse placenta weight. Levels of (**D**) serum cholesterol, (**E**) triglycerides, (**F**) HDL-C levels, (**G**) LDL-C, and (**H**) lysophosphatidic acid levels, respectively. GD, gestation day; HDL-C, high-density lipoprotein cholesterol; LDL-C, low-density lipoprotein cholesterol. *n* = 5, *P < 0.05, **P < 0.01

There was no difference in maternal body weight, fasting blood glucose, or placental weight between the control and chemerin groups on GD18 (Fig. 1A-C), nor did maternal body weight correlate with serum chemerin (Additional file 1 Supplementary Fig. 2A). Serum cholesterol was increased in the chemerin-overexpressing mice (Fig. 1D), while the triglyceride level showed no change (Fig. 1E). Serum HDL-C, LDL-C and LPA were also higher in the chemerin-overexpressing group than in control mice (Fig. 1F-H).

### Overexpression of chemerin in trophoblasts induces lipid accumulation in the mouse placenta

**Fig. 2:**
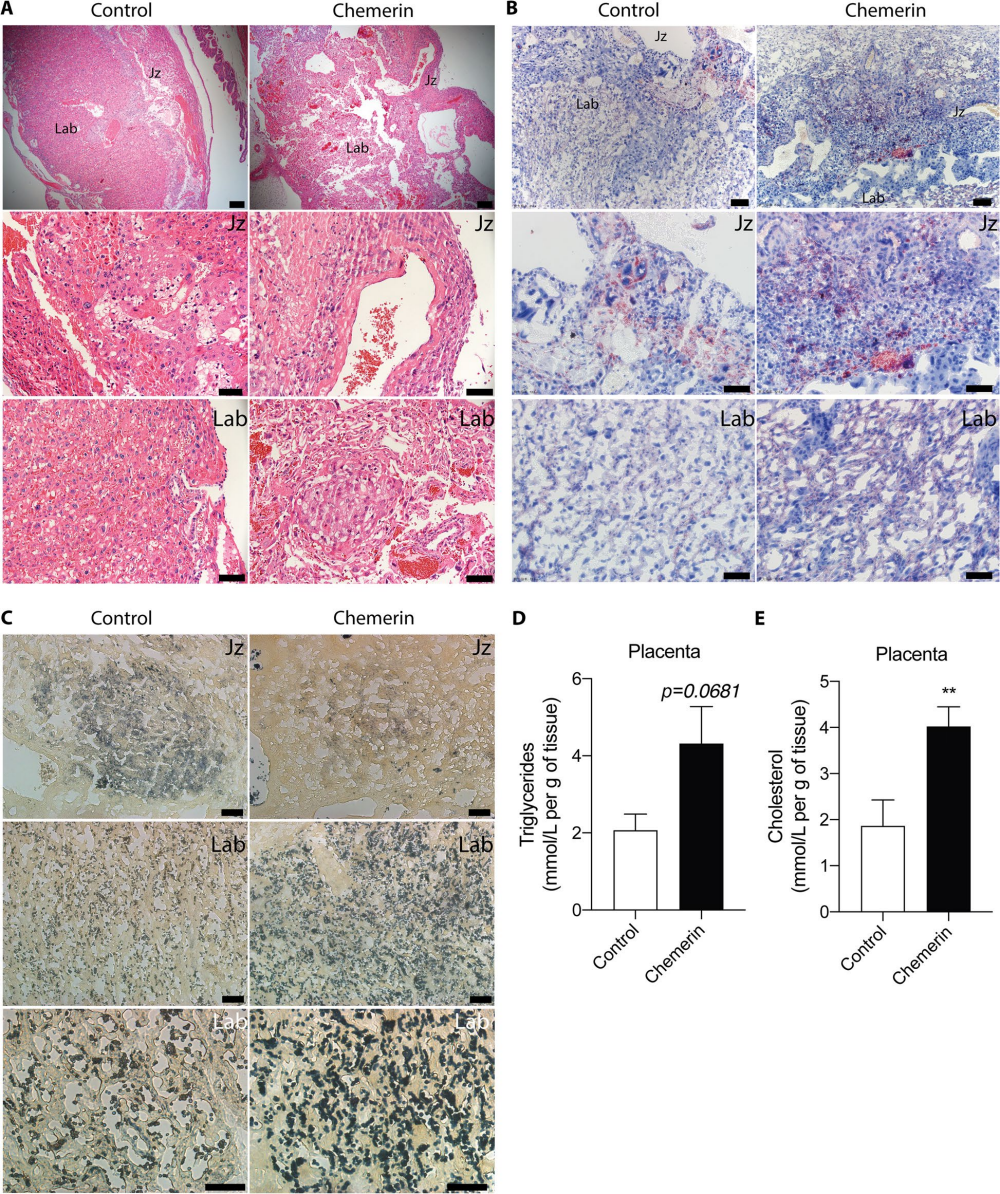
Effect of overexpression of trophoblast-specific chemerin on mouse placental lipid levels. Representative placental images of (**A**) hematoxylin and eosin staining (H&E, top panel: scale bar = 200 μm; Jz and Lab images: scale bar = 50 μm), (**B**) Oil Red O staining (top panel: scale bar = 200 μm; Jz and Lab images: scale bar = 50 μm), and (**C**) PPD staining (Bottom panel of Lab: scale bar = 20 μm; Jz and Lab images: scale bar = 50 μm), respectively. (**D** and **E**) Levels of (**D**) placental triglycerides and (**E**) cholesterol levels, respectively. *n* = 5, **P < 0.01. Jz, junctional zone; Lab, labyrinth zone; PPD, paraphenylenediamine

As shown in Fig. 2A, the placental layers were disorganized in mice with chemerin-overexpression in trophoblasts, and the majority of the trophoblasts was distributed in the labyrinth and junctional zones. Lipid accumulation was observed in the junctional zone and labyrinth of the mouse placenta by Oil Red O staining, and this was increased in mice of the chemerin group (Fig. 2B). The phospholipids, the primary initial source of LPA, were assessed in the placenta by PPD staining. The phospholipid signals were mainly located in the junctional zone and labyrinth. Staining in the labyrinth was stronger than in the junctional zone when chemerin was specifically overexpressed in mouse placentas (Fig. 2C). Furthermore, overexpression of chemerin increased the levels of triglycerides (Fig. 2D) and cholesterol in the placenta (Fig. 2E).

### Overexpression of chemerin in trophoblasts increases the expression of lipid-related proteins through the CMKLR1/CCRL2 axis

**Fig. 3:**
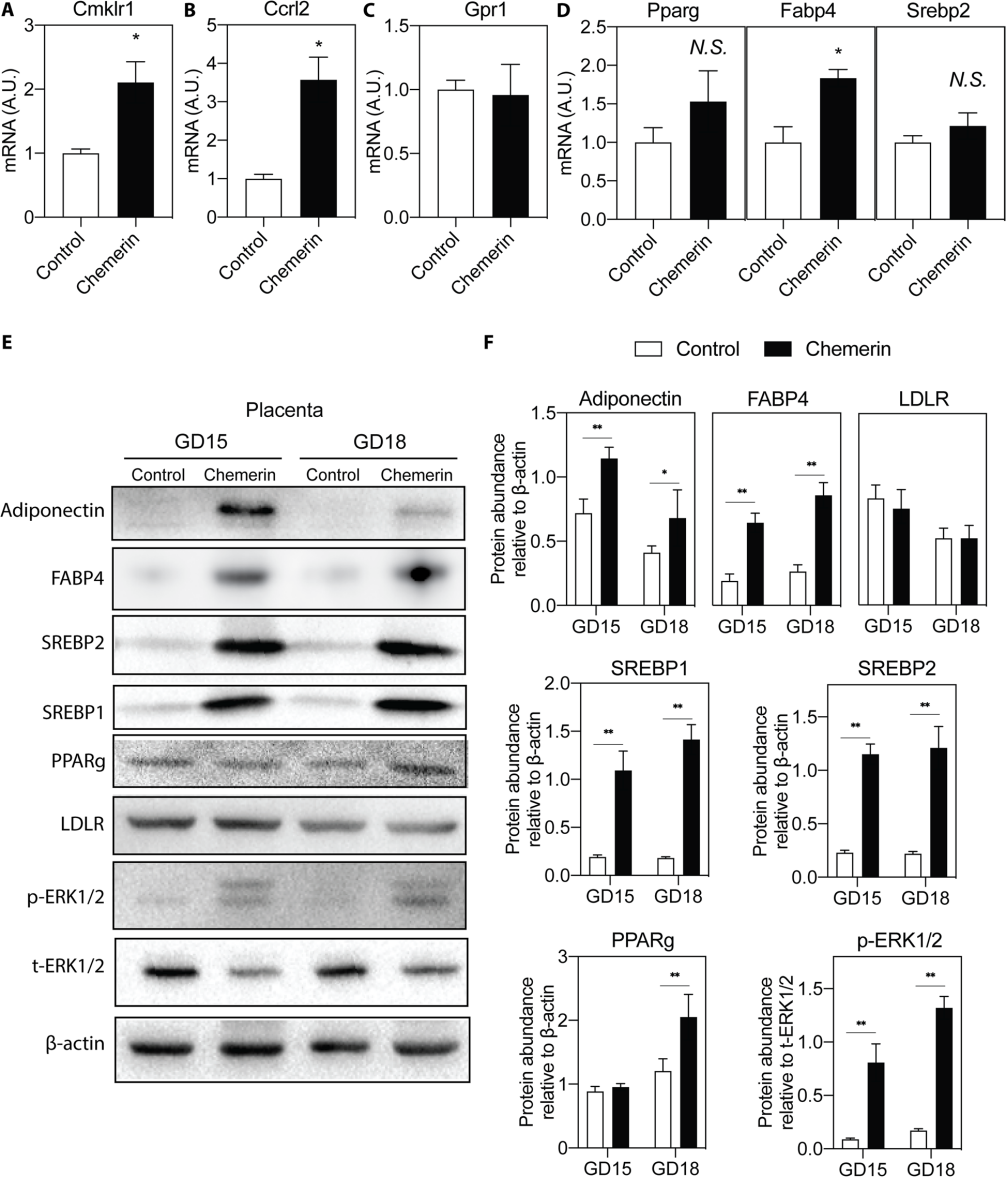
Effect of chemerin overexpression on placental lipid metabolism. The mRNA expression of (**A**) *Cmklr1*, (**B**) *Ccrl2*, (**C**) *Gpr1*, (**D**) *Pparg*, *Fabp4*, and *Srebp2* in placenta of mice at GD18. n = 5, *P < 0.05. **E** The expression of lipid metabolism-related proteins in placenta of mice at GD15 and GD18 by Western blot analysis. **F** The protein abundance was quantified and normalized to the level of β-actin or total ERK1/2. *n* = 5, *P < 0.05, **P < 0.01. A.U.: arbitrary units

CMKLR1, GPR1, and CCRL2 are the three chemerin receptors. The mRNA expression of CMKLR1 and CCRL2, but not GPR1, showed a significant increase in the placentas of mice with chemerin overexpression (Fig. 3A-C). Notably, the expression of lipid-related genes *Pparg, Fabp4*, and *Srebp2* also increased in the chemerin over-expressing placentas (Fig. 3D). The protein levels showed a similar increase (Fig. 3E and F). Furthermore, the protein levels of adiponectin, SREBP1, and the p-ERK1/2 / total ERK1/2 ratio were increased in the placentas of the chemerin group (Fig. 3E and F). However, the protein levels of LDLR were similar in the control and chemerin groups (Fig. 3E and F). The levels of lipid metabolism-related proteins in each group from GD15 and GD18 also did not differ (Fig. 3F).

**Fig. 4:**
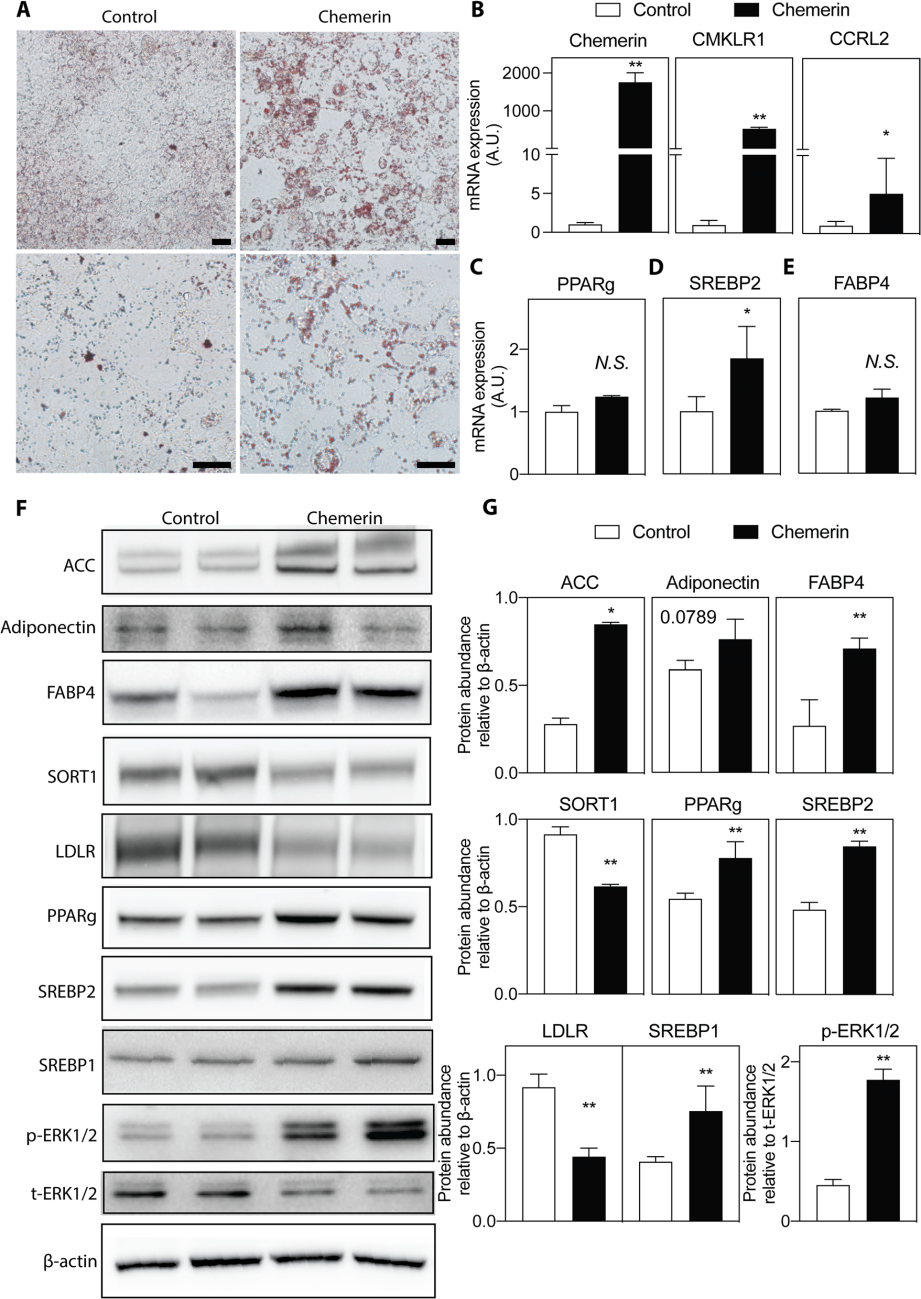
Effect of chemerin overexpression on lipid metabolism in HTR-8/SVneo cells. **A** Representative images of Oil Red O staining of HTR-8/SVneo cells (up panel: scale bar = 200 μm; Bottom panel: scale bar = 50 μm). The mRNA expression of (**B**) Chemerin and its receptors (*GRP1* was not detected), (**C**) *PPARg*, (**D**) *SREBP2*, and (**E**) *FABP4* in HTR-8/SVneo cells. *n* = 3, *P < 0.05. (**F**) Analysis of lipid metabolism in HTR-8/SVneo cells by using Western blot. **G** The protein abundance was quantified and normalized to the level of β-actin or total ERK1/2. *n* = 3 per group; *P < 0.05, **P < 0.01

Accumulation of lipid droplets in HTR-8/SVneo cells increased after chemerin over-expression (Fig. 4A). Chemerin overexpression upregulated the mRNA expression of CMKLR1 and CCRL2, the mRNA and protein levels of PPARg, FABP4, and SREBP2, and the protein level of ACC (Fig. 4B-G). GPR1 could not be detected. Similar to what was observed in the chemerin-overexpressing placenta in vivo, adiponectin and SREBP1 protein levels and the p-ERK1/2 / total ERK1/2 ratio were increased (Fig. 4E and F). However, chemerin overexpression in HTR-8/SVneo cells decreased the protein levels of LDLR and its related protein SORT1 (Fig. 4E and F).

### Overexpression of chemerin in trophoblasts leads to increased production of lysophospholipids and phospholipids

**Fig. 5:**
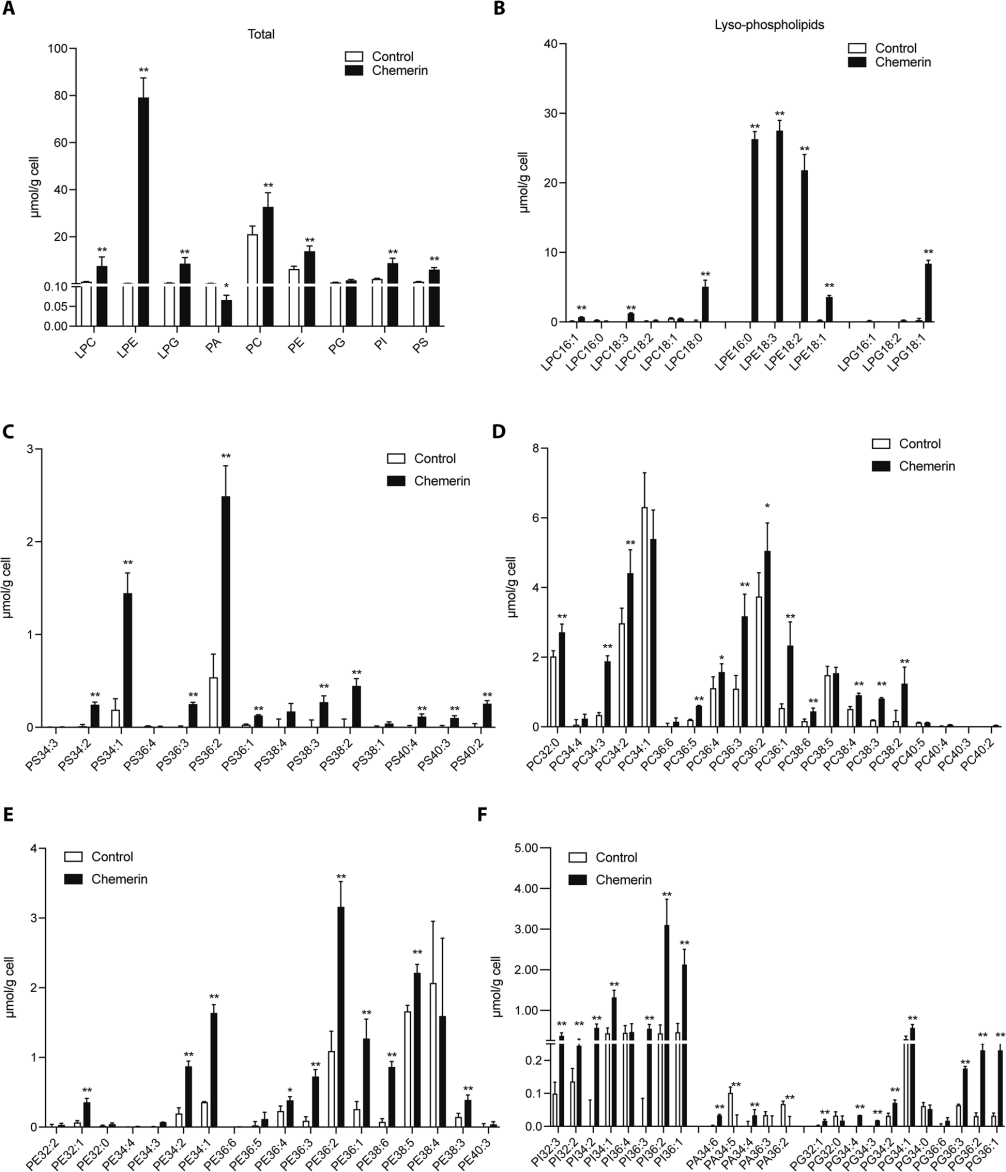
Phospholipidomics analysis of chemerin overexpression in HTR-8/SVneo cells. **A** Total levels of phospholipid classes in chemerin-overexpressing HTR-8/SVneo cells versus control cells. Contents of phospholipid molecular species for (**B**) LPC, LPE and LPG, (**C**) PS, (**D**) PC, (**E**) PE, and (**F**) PA, PG and PI in chemerin-overexpressing HTR-8/SVneo cells or control cells. LPC, lysophosphatidylcholine; LPE, lysophosphatidylethanolamine; LPG, lyso phosphatidylglycerol; PA: phosphatidic acid; PC, phosphatidylcholine; PE: phosphatidylethanolamine; PG: phosphatidylglycerol; PI: phosphatidylinositol; PS: phosphatidylserine. *n* = 3 per group; *P < 0.05, **P < 0.01

The results of phospholipidomics in HTR-8/SVneo cells revealed that the chemerin group produced significantly more lysophospholipids, specifically LPC, LPE, and LPG (Fig. 5A). Additionally, PA decreased while PC, PE, PG, PI, and PS were all increased in the chemerin group (Fig. 5A). More specifically, the levels of LPC species (16:1, 18:0 and 18:3), LPE species (16:0, 18:1, 18:2 and 18:3), and LPG 18:1 were increased in the chemerin group (Fig. 5B). Levels, PS species (34:1, 34:2, 36:1, 36:2, 36:3, 38:2, 38:3, 40:2, 40:3 and 40:4) were increased in the chemerin group (Fig. 5C). Levels of PC species (32:0, 34:2, 34:3, 36:1, 36:2, 36:3, 36:4, 36:5, 38:2, 38:3, 38:4, and 38:6) were increased in the chemerin group (Fig. 5D). Levels of PE species (32:1, 34:1, 34:2, 36:1, 36:2, 36:3, 36:4, 38:3, 38:5 and 38:6) were increased in the chemerin group (Fig. 5E). Levels of PI species (32:2, 32:3, 34:1, 34:2, 36:1, 36:2 and 36:3) were increased in the chemerin group (Fig. 5F). Levels of PA species (34:6 and 34:4) and PG species (32:1, 34:1, 34:2, 34:3, 34:4, 36:1, 36:2 and 36:3) were increased in the chemerin group (Fig. 5F).

## Discussion

The present study disclosed that specific over-expression of chemerin in trophoblasts elevated the lipid levels in mouse maternal serum and resulted in placental lipid accumulation. Overexpression of chemerin also facilitated lipid accumulation in human trophoblastic HTR-8/SVneo cells. Our previous research has reported that specific overexpression of chemerin in trophoblasts causes preeclampsia-like symptoms, and that more chemerin is released from human preeclamptic placentas compared to normal placentas [12].

Chemerin was initially discovered in adipocytes and liver, and has been associated with obesity, metabolic disorders, and cardiovascular disease [10, 11, 22–24]. Chemerin is secreted as pro-chemerin and subsequently activated or inactivated by many proteases, although a full understanding has not yet been established [10, 11, 14, 23, 24]. Accumulating knowledge confirms that chemerin promotes adipogenesis [25–28]. It is well known that chemerin stimulates lipid accumulation in multiple cells, including 3T3-L1 cells and HepG2 cells [25, 26]. Adipocyte differentiation and adipose tissue expansion were impaired when reducing chemerin or blocking its receptor CMKLR1 [27, 28].

Placental accumulation of lipids may contribute to pregnancy-related disorders, such as preeclampsia and gestational diabetes. In gestational diabetes, BMI is a major risk factor [29], while in preeclampsia, though BMI is a risk factor, inflammation is considered the main trigger for dyslipidemia [30]. Indeed, around 80% of preeclampsia cases occurred at a normal BMI [31], and we previously reported a higher placental chemerin release in preeclamptic patients with a normal BMI [12]. Moreover, a rat preeclampsia model involving intraperitoneal injection of N^ω^-nitro-l-arginine methyl ester (L-NAME; a nitric oxide synthase inhibitor) was characterized by placental inflammation and lipid accumulation [32], confirming that inflammation might be an additional factor contributing to lipid metabolism in the placenta [12].

In the present study, the elevated expression of CMKLR1 and CCRL2 in chemerin-overexpressing trophoblasts suggests that these receptors are involved in the effect of chemerin on lipid accumulation during pregnancy. The lipid elevation concerns cholesterol rather than triglycerides, although the latter correlate positively with chemerin in metabolic disorders [33, 34]. Both in vivo and in vitro studies have shown that chemerin promotes lipid uptake and storage, evidenced by the enhanced production of PPARg [35], adiponectin [36], p-ERK1/2 [37–39], and FABP4 [40]. In HTR-8/SVneo trophoblast cells, chemerin increased cholesterol biosynthesis by upregulating SREBP2 [41], while it diminished LDL endocytosis via downregulation of both the LDLR and SORT1 [42, 43]. This subsequently reduced LDL uptake and increased cholesterol release. As the SREBP2 target gene, LDLR is supposed to increase with the upregulation of SREBP2, but chemerin is also reported as the SREBP2 response gene that might competitively affect LDLR expression [44]. Moreover, chemerin overexpression upregulated the tricarboxylic acid (TCA) cycle and fatty acid synthesis by increasing ACC and SREBP1 [45, 46], which further enhances lipid deposition. In humans, PPARg levels increase during normal gestation and unaltered in preeclampsia, although incubating trophoblasts with serum from preeclamptic women did upregulate PPARg [47, 48]. Circulating and placental adiponectin is increased in early (but not late) pregnancy [49, 50], while in preeclampsia, plasma adiponectin is higher than in healthy control [51]. Placental FABP4 levels are upregulated in preeclamptic women [32, 52]. Placental SREBP1-c and p-ERK1/2 are increased in preeclamptic patients [53, 54]. Placental LDLR expression did not differ between preeclamptic and control pregnant women [55]. In the extravillous cytotrophoblasts, SORT1 is decreased in severe preeclampsia compared to normal pregnancy [56].

Both placental lipids and the maternal blood lipidome may be involved in the pathophysiology of severe preeclampsia [8]. The total phospholipid content is increased in preeclamptic placental tissue [57, 58]. In this study, chemerin overexpression increased the levels of phospholipids, lysophospholipids, and cholesterol in maternal blood and the placenta, with particular accumulation in the labyrinth layer. These changes might induce lipoprotein dysfunction. Oxidation products (e.g., oxLDL) derived from cholesterol and lysophospholipids contribute to the pathogenesis of preeclampsia and cause oxidative stress, eNOS dysfunction, endothelial dysfunction, and acute atherosclerosis [59]. Normal HDL has been described as an atheroprotective particle, but in patients with cardiovascular disease, HDL can be converted to a dysfunctional form and exhibit proatherogenic effects [60]. Dysfunctional HDL is characterized by oxidized phospholipids and lysophospholipids, thereby losing its ability to promote cholesterol efflux and prevent LDL oxidation [61]. LPCs associate with liver injury, kidney injury, and inflammation [62–64]. In vitro, LPC 16:0 and LPC 18:0 promote lipid accumulation and apoptosis [62, 63]. LPE 18:2 activates G-protein-coupled receptor signaling and increases lipid accumulation [65, 66], while serum LPE 16:0 associates with diabetes mellitus [67]. LPG is the agonist of G-protein-coupled receptor 55 and induces inflammation in macrophages [68]. The phospholipids PC, PS, PI, and PE are elevated in the preeclamptic placenta [58]. The phospholipidomic findings in human trophoblasts demonstrate that chemerin enhances the contents of the lysophospholipid molecular species of LPC 16:1 and 18:2, and LPE18:2. Similar alterations have been reported in blood of the preeclamptic women [8, 69]. Additionally, chemerin overexpression enhanced the LPC 16:0, PE 34:2, and PE 34:3 levels, which also occurs in preeclamptic placentas [70]. Though the serum LPA level was significantly higher in the chemerin group, such changes were not found in HTR-8/SVneo cells. This might be due to the relatively low activity of lysophospholipase D in this cell line [71]. LPA is mainly produced from LPC, LPE, or LPG through the activity of lysophospholipase D and then released into the circulation [72, 73]. However, it is notable that the total PA levels were decreased in the chemerin-overexpressing cells.

**Fig. 6:**
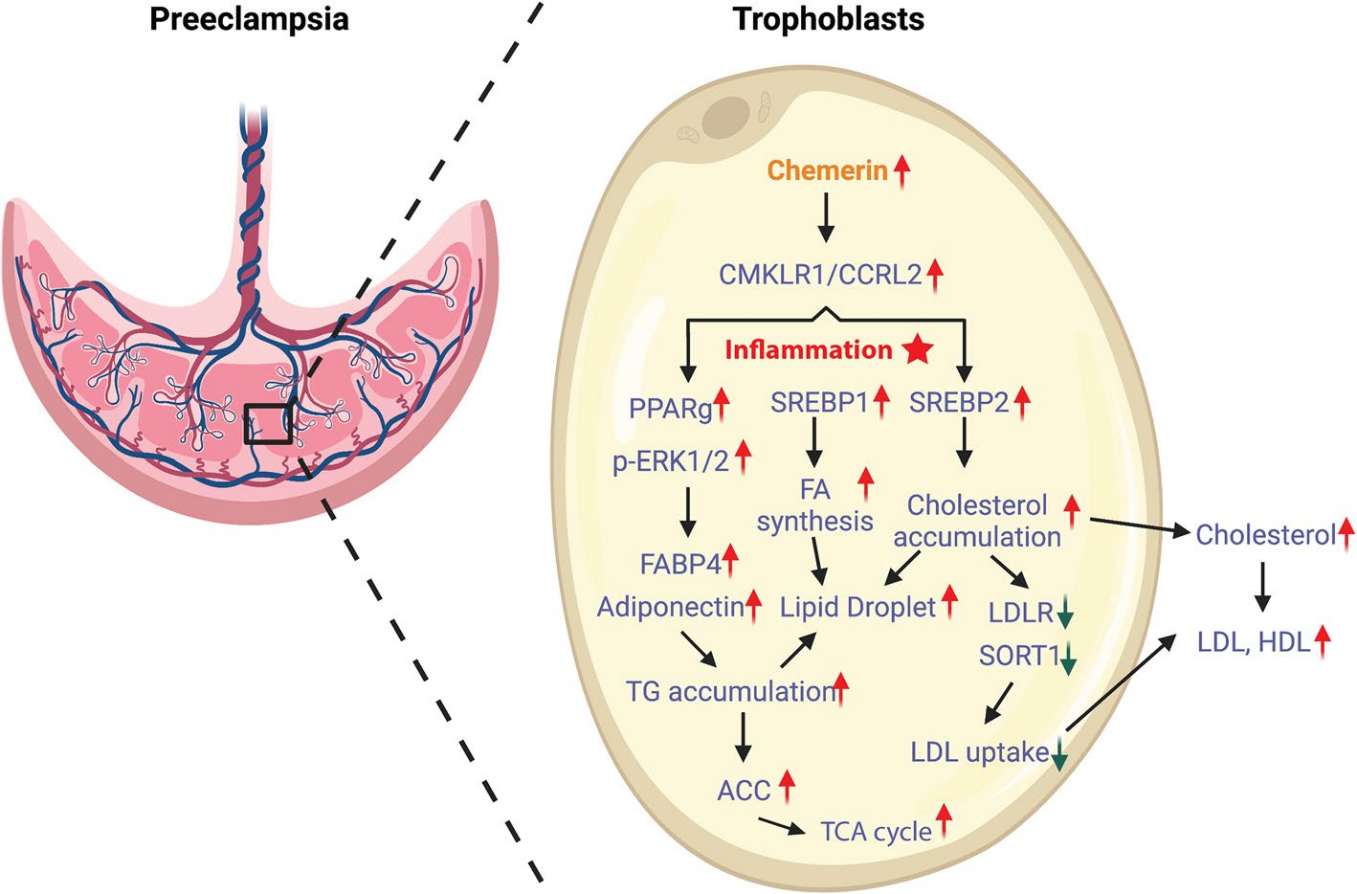
Schematic view of roles of chemerin in preeclampsia dyslipidemia. In preeclampsia condition, a high level of chemerin is released from trophoblasts in the placenta, inducing an inflammatory condition and further increasing the levels of placental lipids (TG, Chol, and phospholipids), lipid droplet accumulation, and the TCA cycle. Meanwhile, chemerin inhibits LDL uptake by reducing LDLR and SORT1 in trophoblasts, which leads to an increase in the release of lipids and the lipid-related protein (TG, Chol, phospholipids, and chemerin) from the placenta to maternal circulation, as well as a lower LDL uptake from circulation to placenta, eventually resulting in dyslipidemia in the patient (This figure with the credit “Created with BioRender.com.”)

In conclusion, the present study confirms that a preeclampsia condition can be obtained in mice when overexpressing chemerin in trophoblasts. This induces an inflammatory condition and upregulates the content of placental lipids (triglycerides, cholesterol, and phospholipids), lipid droplet accumulation, and the TCA cycle. Meanwhile, chemerin inhibits LDL uptake by reducing LDLR and SORT1 in trophoblasts, which leads to an increase in the release of lipids and the lipid-related protein (triglycerides, cholesterol, phospholipids, and chemerin) from the placenta to the maternal circulation, as well as a lower LDL uptake from the circulation to the placenta, eventually resulting in dyslipidemia in the patient (Fig. 6). Future studies are needed to sort out whether reducing inflammation or inhibiting chemerin receptors might attenuate dyslipidemia in the placenta and circulation of pregnant women.

### Comparisons with other studies and what does the current work add to the existing knowledge

Many studies have studied lipid changes in preeclampsia by comparing blood and placental tissue from healthy pregnant women and women with preeclampsia [57, 58]. The underlying mechanism remained unknown. Given that adipokine chemerin plays an important role in the lipid metabolism of adipocytes and hepatocytes, we now present data showing that placental overexpression of chemerin might be causally involved in lipid dysregulation in preeclampsia. This offers new pharmacological targets to treat this condition.

### Study strength and limitations

Building on a previously developed preeclampsia mouse model, based on placenta-specific overexpression of the adipokine chemerin, we were able to link chemerin to lipid accumulation in the junctional zone and labyrinth in the placenta, as well as to lipid accumulation in the circulation. Further mechanistic insights were obtained from chemerin-overexpressing human placental trophoblasts cells. Although this is a unique combination of in vivo and in vitro observations, to what degree this is also true in human preeclampsia remains to be determined. Furthermore, it is difficult to precisely dose chemerin expression via the lentiviral system. Due to a lack of samples, phospholipidomics analysis was not possible in the placenta. We also did not analyze the precise consequences of maternal HDL-C elevation.

## Conclusions

In conclusion, this study suggests that chemerin disturbs trophoblast lipid metabolism by increasing lipid uptake, lipid droplet deposition, cholesterol biosynthesis, the TCA cycle and by inhibiting LDL endocytosis. A lipidome analysis in human placentas with high expression of chemerin may help to unravel the role of chemerin in the pathogenesis of preeclampsia. As a protein with multifaceted effects on lipid metabolism, angiogenesis, and inflammation, chemerin may represent a potential therapeutic target for the treatment of preeclampsia.

## Supplementary Information

**Additional file 1.** Supplementary Materials

**Additional file 2.**

**Additional file 3.**

## Data Availability

The data of the current study are available from the corresponding author on reasonable request.

## Abbreviations

CCRL2: C-C chemokine receptor-like 2
CMKLR1: Chemokine like receptor 1
ELISA: Enzyme-linked immunosorbent assay
ERK: Extracellular signal-regulated protein kinase
GD: gestational day
GPR1: G protein-coupled receptor 1
HDL-C: High-density lipoprotein cholesterol
LDL-C: Low-density lipoprotein cholesterol
LPC: Lysophosphatidylcholine
LPE: Lysophosphatidylethanolamine
LPG: Lyso phosphatidylglycerol
PA: Phosphatidic acid
PC: Phosphatidylcholine
PE: Phosphatidylethanolamine
PFA: Paraformaldehyde
PG: Phosphatidylglycerol
PI: Phosphatidylinositol
PPD: Paraphenylenediamine
PS: Phosphatidylserine
qRT-PCR: Quantitative reverse transcription-polymerase chain reaction
TCA: The tricarboxylic acid
TG: Triglyceride
SDS-PAGE: sodium dodecyl-sulfate polyacrylamide gel electrophoresis
FABP4: fatty acid binding protein 4; PPARg: peroxisome proliferator activated receptor gamma
ACC: Acetyl-CoA carboxylase
LDLR: low density lipoprotein receptor
SREBP2: sterol regulatory element-binding protein 2
SREBP1: sterol regulatory element-binding protein 1
SORT1: sortilin 1
MTBE: methyl-tert-butyl ether
L-NAME: Nω-nitro-l-arginine methyl ester.
