# Supplementary material for: Adipokine chemerin overexpression in trophoblasts leads to dyslipidemia in pregnant mice: implications for preeclampsia
Source: Lipids Health Dis. 2023 Jan 25;22:12. doi: 10.1186/s12944-023-01777-4 (PMC9875463; doi:10.1186/s12944-023-01777-4)
Supplement: Supplementary file 1 — Additional file 1. Supplementary Materials [file 12944_2023_1777_MOESM1_ESM.docx]

**Supplemental Materials**

**Table S1.** Primers used for qPCR.

| Gene | Primer Sequence |
| --- | --- |
| *Mouse β-actin* | 5’TGCGGCCGCCTGTCTAGGGCTTATTTG3’; 5’GCAGTACATAATTTACACAGAAGCAAT3’ |
| *Mouse Chemerin* | 5’TACAGGTGGCTCTGGAGGAGTTC3’; 5’CTTCTCCCGTTTGGTTTGATTG3’ |
| *Mouse Cmklr1* | 5’CGAGTTCTCAAACCCTGAAGTCGC3’; 5’CAAGTCCACAAAGTAGCCAAAGCC3’ |
| *Mouse Ccrl2* | 5’CTCTGCTTGTCCTCGTGCTT3’; 5’GCCCACTGTTGTCCAGGTAG3’ |
| *Mouse Gpr1* | 5’GGAGCTCAGCATTCATCACA3’; 5’GACAGGCTCTTGGTTTCAGC3’ |
| *Mouse Pparg* | 5’TCTGGGAGATTCTCCTGTTGA3’; 5’GGTGGGCCAGAATGGCATCT3’ |
| *Mouse Fabp4* | 5’TGAAATCACCGCAGACGACAGG3’; 5’GCTTGTCACCATCTCGTTTTCTC3’ |
| *Mouse Srebp2* | 5’GACCGCTCTCGAATCCTCTTATGTG3’; 5’GTTTGTAGGTTGGCAGCAGCA3’ |
| *Human β-actin* | 5’AGCGAGCATCCCCCAAAGTT3’; 5’GGGCACGAAGGCTCATCATT3’ |
| *Human PPARg* | 5’AGCCTGCGAAAGCCTTTTGGTG3’; 5’GGCTTCACATTCAGCAAACCTGG3’ |
| *Human SREBP2* | 5’CTCCATTGACTCTGAGCCAGGA3’; 5’GAATCCGTGAGCGGTCTACCAT3’ |
| *Human FABP4* | 5’ACGAGAGGATGATAAACTGGTGG3’; 5’GCGAACTTCAGTCCAGGTCAAC3’ |
|  |  |
|  |  |
|  |  |


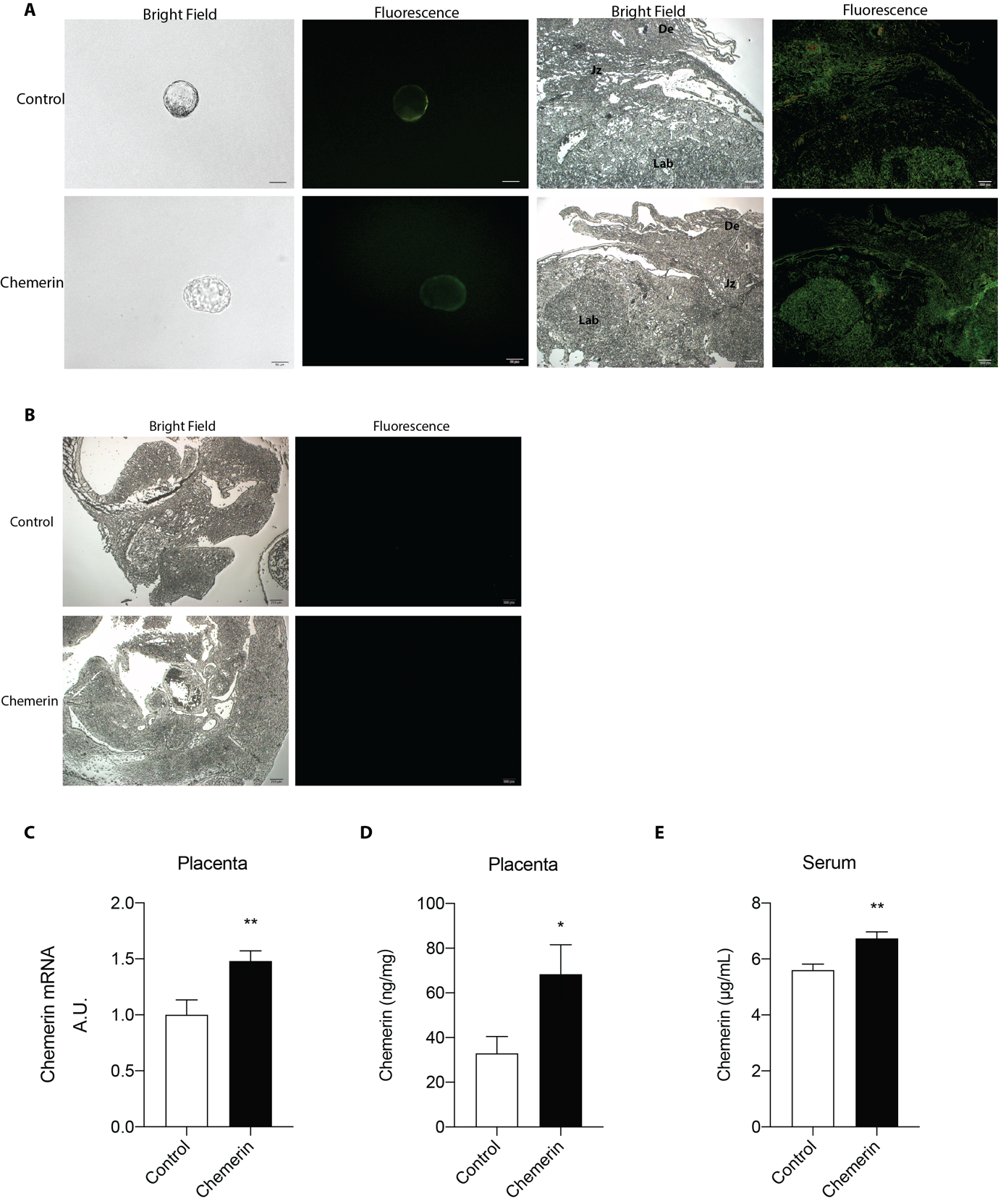


**Figure S1**. **Verification of trophoblast-specific chemerin overexpression.** (**A**) The blastocysts transduced with lentiviruses expressing GFP alone (Control) or chemerin+GFP (Chemerin), GFP is only observed in the trophectoderm (outer layer, scale bar represents 50 μm), while during pregnancy GFP is expressed in the junction zones and labyrinth layers of the mouse placenta at gestational day 18 (scale bar represents 200 μm). (**B**) GFP cannot observed in the fetus at gestational day 18 (scale bar represents 200 μm). Chemerin overexpression is verified in the chemerin group on placental mRNA (**C**), placental protein (**D**), and serum levels (**E**). N=5, **P*<0.05, ***P*<0.01. A.U.: arbitrary units.


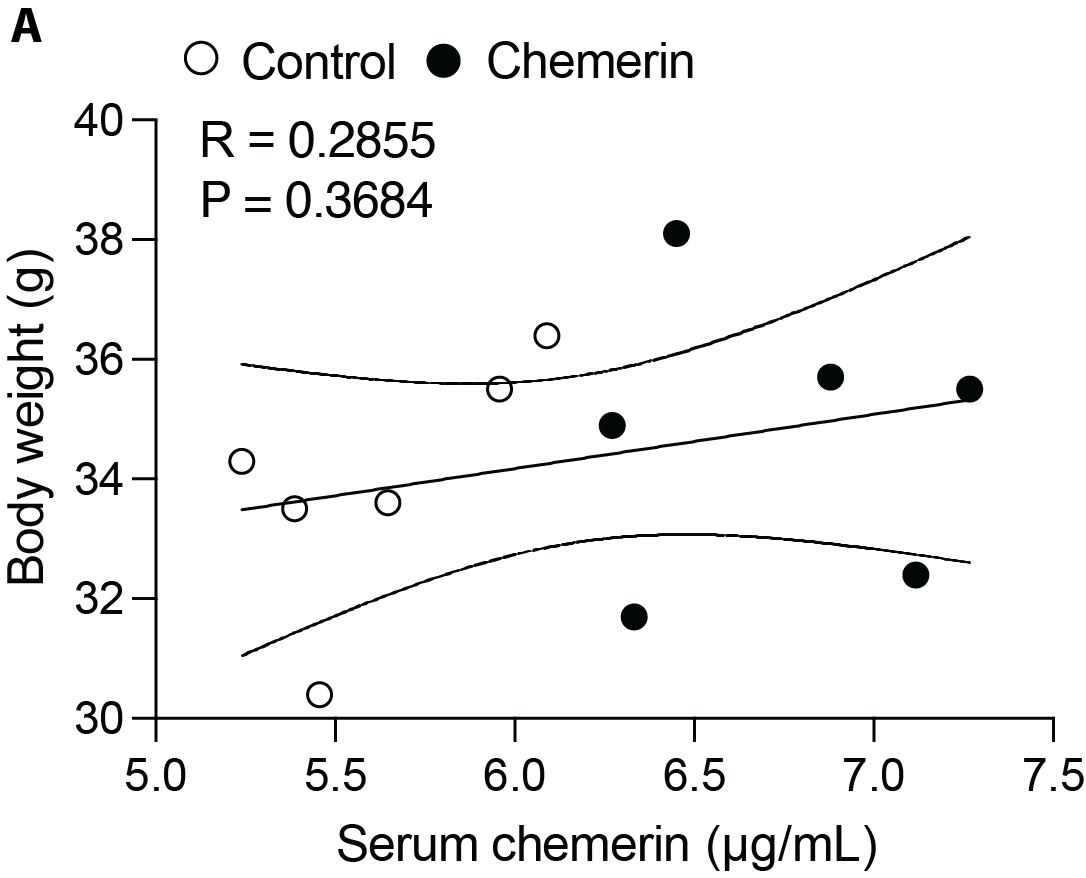


**Figure S2**. **The correlation of maternal body weight and serum chemerin.** (**A**) Serum chemerin levels showed no significant correlation to maternal body weight. N=5.
