# Supplementary material for: Adipokine chemerin overexpression in trophoblasts leads to dyslipidemia in pregnant mice: implications for preeclampsia
Source: Lipids Health Dis. 2023 Jan 25;22:12. doi: 10.1186/s12944-023-01777-4 (PMC9875463; doi:10.1186/s12944-023-01777-4)
Supplement: Supplementary file 2 — Additional file 2. [file 12944_2023_1777_MOESM2_ESM.pdf]

Proofread My Essay ZiboYimore Translation CO. LTD

13/12/2022

To whom it may concern:

The purpose of this letter is to verify that Zibo Yimore Translation CO. LTD provided the English proofreading services for the following manuscript: Adipokine chemerin overexpression leads to dyslipidemia in preeclampsia-like mice.

Author: Lunbo Tan, Zijun Ouyang, Zhilong Chen, Fen Sun, Haichun Guo, Feng Wang, Monique Mulder, Yuan Sun, Xifeng Lu, Jian V. Zhang, A.H. Jan Danser, Koen Verdonk, Xiujun Fan and Qing Yang.

The basic language editing including the correction of grammar, punctuation and syntax was performed normatively with our best efforts. And the edited document was returned to the writer on 13/12/2022. We are unaware of any changes or additions made to the manuscript after that time. We have kept the specimen of the received original file in case of any legal dispute.

Sincerely,

Henry Graff

Zibo Luyi Translation CO. LTD

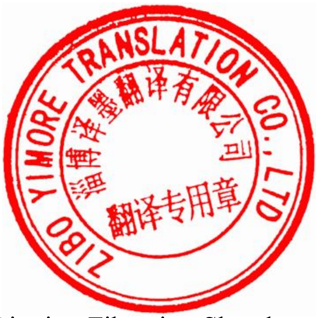

Tel: 0533-2775538

Address: Meishistreet NO.137, Zhangdian District, Zibo city, Shandong Province
