# Supplementary material for: Adipokine chemerin overexpression in trophoblasts leads to dyslipidemia in pregnant mice: implications for preeclampsia
Source: Lipids Health Dis. 2023 Jan 25;22:12. doi: 10.1186/s12944-023-01777-4 (PMC9875463; doi:10.1186/s12944-023-01777-4)
Supplement: Supplementary file 3 — Additional file 3. [file 12944_2023_1777_MOESM3_ESM.pdf]

**Abstract**

**Background:** Adipokine chemerin regulates adipogenesis and the metabolic function of both adipocytes and liver which is elevated in circulation and placenta in preeclamptic women. Our previous research reported that overexpression of chemerin in placental trophoblasts induced preeclampsia-like symptoms in mice. Preeclampsia is known to be accompanied by dyslipidemia, albeit via unknown mechanisms. Herein, we hypothesized that chemerin might be a potential biomarker of dyslipidemia.

**Methods:** Circulating lipid markers including cholesterol, triglycerides, high-density lipoprotein cholesterol (HDL-C), lysophosphatidic acid, and low-density lipoprotein cholesterol (LDL-C) were detected in the preeclampsia-like mouse with chemerin overexpression in placental trophoblasts. Histomorphology and lipid accumulation in placenta were also detected by using hematoxylin and eosin staining, paraphenylenediamine staining as well Oil Red O staining. Expressions of lipid metabolism related genes and proteins were detected in placenta and the chemerin-overexpressed human trophoblasts. In addition, phospholipidomics analysis was performed to detect phospholipid levels in chemerin-overexpressed trophoblasts.

**Results:** Overexpression of chemerin in trophoblast increased the circulating and placental levels of cholesterol rather than triglycerides. It also increased the seral levels of lysophosphatidic acid, HDL-C, and LDL-C, and induced placental lipid accumulation. Mechanistically, chemerin upregulated the levels of peroxisome proliferator-activated receptor Y, fatty acid-binding protein 4, adiponectin, sterol regulatory element-binding protein 1 and 2, and the ratio of phosphorylated extracellular signal-regulated protein kinase (ERK)/total ERK in the placenta of mice and human trophoblasts. Furthermore, chemerin

overexpression in human trophoblasts increased the production of lysophospholipids and phospholipids, particularly lysophosphatidylethanolamine.

**Conclusions:** Overexpression of placental chemerin production disrupts trophoblast lipid metabolism via the CMKLR1/CCRL2 axis, thereby potentially contributing to dyslipidemia in preeclampsia.

**Keywords:** chemerin; preeclampsia; dyslipidemia; placenta; trophoblast; phospholipids

## Background

Preeclampsia is a progressive, multi-systemic disorder which causes a high prevalence of pregnancy-related morbidity and mortality [1]. Clinically, preeclampsia appears after 20 weeks of gestation with de novo hypertension and either proteinuria or hemolysis, elevated liver enzymes and low platelets (HELLP) syndrome [2, 3]. The exact pathophysiology is still unknown. Increasing evidence reveals an association between this pathological condition and an imbalance in lipid regulation. Previous studies revealed that preeclamptic patients have a higher seral level of triglycerides, total cholesterol, phospholipids and low-density lipoprotein cholesterol (LDL-C) [4-9].

Chemerin, a small chemotactic adipokine, affects blood pressure, cholesterol levels, adipose tissue functions, and insulin sensitivity [10]. It functions through three receptors, C-C motif chemokine receptor-like 2 (CCRL2), chemerin receptor 1 (CMKLR1), chemerin receptor 2 (GPR1) [11, 12]. Blood chemerin levels are elevated in preeclampsia patients and correlate positively with preeclampsia severity [4, 13-15]. Our previous work, we developed a preeclampsia-like mouse model which was induced by specific overexpression of chemerin in placental trophoblasts. Severe placental vascular damage, significant embryonic growth restriction and lethality were observed in this animal model [16]. The data indicates that chemerin originates, at least partialy, from placenta during pregnancy in mice.

We hypothesized that a high level of chemerin released from placental trophoblasts might be a risk factor contributing to dyslipidemia during preeclamptic conditions. In the current study, we further investigated lipid accumulation in placenta tissues in this mouse model, and phospholipid profile was also explored in chemerin-everexpressed human trophoblast cells.

## Materials and Methods

**Production of lentiviral vectors**

The construction of lentiviral vectors and the chemerin overexpression model has been described in our previous work [16]. In brief, LV-mChemerin-GFP and LV-hChemerin-GFP were generated by cloning mouse chemerin or human chemerin into the lenti-viral vector respectively (LV-GFP, System Biosciences, USA). The method for producing lentiviral particles was previously described [16-18]. The titer of lentivirus was measured by using a comercial kit (Cell Biolabs Inc, San Diego, CA, USA).

**Preparation of preeclampsia mice model**

Animal experiments were conducted at the Shenzhen Institutes of Advanced Technology in compliance with the Chinese Academy of Sciences board approval. CD-1 mice at the age of 8-10 weeks were purchased from Beijing Vital River Laboratory Animal Technology Co., Ltd. (Beijing, China). The procedures for generating the preeclampsia mouse model were previously described [16-18]. The vaginal plug day was marked as the first day of pregnancy or pseudopregnancy (gestational day 1; GD1). On GD4, zona-free blastocysts were transferred to GD3 pseudopregnant mice after 6 h-incubation with LV-mChemerin-GFP (Chemerin) or LV-GFP (Control). Placentas and blood samples were collected. On GD15 and GD18, mice were anaesthetied with 5% isoflurane administration and cervical dislocation. Placentas were collected for histopathologyical diagnosis as well as mRNA and protein analyses. Serum samples were prepared for biochemical tests.

**Biochemical measurements**

On GD18, bloods were sampled from the mouse tail for fasting blood glucose measurement by a glucose meter (Roche, Basel, Switzerland). The serum lipids levels (included triglyceride, high-density lipoprotein cholesterol (HDL-C), cholesterol, and low-density lipoprotein

cholesterol (LDL-C)) were determined using an automatic biochemical analyzer (Roche, Basel, Switzerland). Placental triglycerides and cholesterol were extracted by Folch's method [19]. ELISA kits were utilized to detect seral levels of lysophosphatidic acid (LPA, BLUEFBIO, Shanghai, China) and chemerin (R&D Systems, Minneapolis, MN, USA) respectively.

**Histology**

Mouse placentas were incubated in 4% paraformaldehyde (PFA) for 24 hours at four degrees Celsius, followed by embedding with paraffin or O.C.T. solution (Sakura, Torrance, CA, USA).

*Oil Red O staining.* Frozen sections of the placenta, 10 µm thick, were fixed in 4% PFA and dehydrated in a gradient of sucrose. Sections were tained in Oil Red O staining solution according to manufacturer's instructions (Sigma-Aldrich).

*Hematoxylin & eosin (H&E) staining.* Five µm thick paraffin slices were obtained. Slides were deparaffinized, and rehydrated, then stained by H&E.

*Phospholipids staining.* After fixation and dehydration, placental frozen sections were stained by paraphenylenediamine (PPD) staining method as described previously [20]. Briefly, 1% PPD (Aladdin, Shanghai, China) was added, and incubated the slides for 10 minutes at 20 °C , then rinsed in 100% ethanol for 5 min. Sections were dried and covered with mounting solution.

The images of statining were captured by an Olympus BX53 microscope (Olympus, Japan).

**Preparation of chemerin-overexpressed trophoblast cell model**

The HTR-8/SVneo cells were kept in DMEM/F12 medium with 10% fetal bovine serum (Hyclone, Logan, UT, USA) at 37°C with 5% CO2, which was received from Dr. Charles H.

Graham at Queen's University. Preparation of the chemerin-overexpressed cells has been described previously [16]. Lipid accumulation was measured in the cells by Oil Red O staining.

**Gene expression analysis**

First, RNA was extracted from the control and chemerin groups in mouse placenta and trophoblast cells using TRIzol® reagent (Invitrogen, Carlsbad, CA). Then, a commercial kit (Invitrogen) used to reverse transcript RNA to DNA templates. Finally, the real-time PCR was performed using an SYBR green-based qPCR kit (TOYOBO, Osaka, Japan) and run on the System (Roche, Pleasanton, CA, USA). Sequences of the specific primers are displayed in the **Supplementary Table 1**. The comparative C(T) method was used to deterimine the mRNA levels of genes which were normalised by using β-actin as an internal reference gene.

**Western blot**

The total proteins of the samples from the control and chemerin groups in mouse placenta and trophoblast cells were extracted from the placental homogenates using RIPA lysis buffer, and quantified using the Bradford method (Thermo Fisher Scientific, Waltham, MA, USA). A total of 20 μg protein of each sample were electroblotted onto PVDF membranes (Millipore, Burlington, MA) after SDS-PAGE separation. The following method has been described previously [16]. Protein signals were developed using an enhanced chemiluminescence kit and observed with a ChemiDoc system (Bio-Rad, Irvine, CA). The primary antibodies and dilutions are listed as following: rabbit polyclonal antibodies against adiponectin (1:500, Proteintech, Wuhan, China), FABP4 (1:1000, Abcam, Cambridge, UK), PPARg (1:1000, Cell Signaling Technology, Boston, MA, USA), ACC (1:1000, Cell Signaling), ERK1/2 (1:1000, Cell Signaling), phospho-ERK1/2 (1:1000, Cell Signaling), LDLR (1:1000, Thermo Fisher), mouse monoclonal antibodies against SREBP2 and SREBP1 (1:1000, Santa Cruz Biotechnology, Santa

Cruz, CA, USA), β-actin (1:5000, Sigma-Aldrich) and SORT1 (1:1000, BD Bioscience, Ann Arbor, MI, USA).

**Phospholipidomics analysis in chemerin-overexpressed cell model**

*Lipid extraction*. Intracellular lipids were extracted by methyl-tert-butyl ether (MTBE) method [21, 22]. The chemerin-overexpressed and control trophoblast cells (3 pooled batches of cells per sample) were first resuspended in 1.5 mL methanol. Then MTBE (5 mL) was added, and the mixtures were vortexed for 1 h at 20°C and culured in water (1.25 mL) for 10 min. After centrifugation, the upper organic lipid phases were collected and dried in a vacuum centrifuge. Then, lipids were dissolved in $CHCl_3$/methanol/water (200 µL, 60:30:4.5 by volumn) followed by phospholipidomics analysis.

*Phospholipids metabolomic analysis*. 150 µL of each lipid fraction was mixture with a 1650 µL of chloroform/methanol/ammonium acetate (300:665:35 by volumn). Phospholipid classes were identified by electrospray ionisation tandem mass spectrometry, and quantified via using the internal standards [23], including 6.6 nmol di-14:0-phosphatidylcholine (PC), 6.6 nmol 13:0- lysophosphatidylcholine (LPC), 6.6 nmol 19:0-LPC, 3.6 nmol di-14:0-phosphatidylethanolamine (PE), 3.6 nmol 14:0-lysophosphatidylethanolamine (LPE), 3.6 nmol 18:0-LPE, 3.6 nmol di-14:0- phosphatidylglycerol (PG), 3.6 nmol 14:0-lysophosphatidylglycerol (LPG), 3.6 nmol 18:0-LPG, 3.6 nmol di14:0- phosphatidic acid (PA), 2.4 nmol di14:0- phosphatidylserine (PS), and 1.63 nmol di18:0- phosphatidylinositol (PI). The Shimadzu UFLC LC/MS system (Triple TOFTM 5600 plus; AB Sciex, Foster City, CA) combined with a C18 column (Kinetex 2.6u C18 100A 150 x 2.1 mm00F-4462-AN) was used for the analysis and identification of the lipid components. The mass spectrometry conditions were: ion source temperature, 300°C; electrospray voltage, +5.5 kV or -4.5 kV; positive ion mode,

collision voltage (CE), PE and LPE, +28 V; PC and LPC, +40 V; negative ion mode, collision voltage (CE), PI, -58 V; PG, LPG and PA, -57 V; PS, -34 V. The data were analyzed qualitatively using Peakview, a companion software provided by AB Sciex, and then quantitatively using MultiQuant software.

**Statistical analysis**

The data represent as mean ± SD. GraphPad Prism was used for statistical analysis (version 8, La Jolla, CA, USA). The data normality was evaluated by the Shapiro-Wilk test. Statistic difference between groups was analyzed by using the Student's t-test, indicating as *$P < 0.05$ and **$P < 0.01$.

## Results

**Overexpression of chemerin in trophoblast increased lipid levels in mice**

The specific overexpression of chemerin was firstly verified in trophoblasts. As shown in Supplementary Fig. 1, GFP signals were observed in blastocyst trophectoderm and placentas of the control or chemerin group mouse on GD18 (Supplementary Fig. 1A), and no GFP signals were detected in the fetus (Supplementary Fig. 1B). The expression of either chemerin mRNA or protein was increased in the chemerin group's placentas (Supplementary Fig. 1C and D). Furthermore, circulating chemerin level was remarkably higher in the chemerin group than in the control group (Supplementary Fig. 1E).

There was no difference in maternal body weight, fasting blood glucose, or placental weight between the control and chemerin groups on GD18 (Fig. 1A-C). Serum cholesterol level was increased in the chemerin-overexpressed mice (Fig. 1D), while the triglyceride level showed no singnificant change (Fig. 1E). Biochemical measurements also revealed that serum

HDL-C, LDL-C and LPA were higher in the chemerin-overexpressed group than those in the control mice (Fig. 1F-H).

**Overexpression of chemerin in trophoblast induced lipid accumulation in mouse placenta**

As shown in Fig. 2A, the placental layers were disorganized in mice with chemerin-overexpression in trophoblasts, and the majority of trophoblasts were distributed in the labyrinth and junctional zones. Lipid accumulation was observed in the junctional zone and labyrinth of the mouse placenta by Oil Red O staining, which was increased in mice of the chemerin group (Fig. 2B). The phospholipids, the primary initial source of LPA, were assessed in the placenta by PPD staining. The phospholipid signals were mainly located in the junctional zone and labyrinth, however staining signals in the labyrinth was stronger than that in the junctional zone when the chemerin was specifically overexpressed in mouse placentas (Fig. 2C). Furthermore, overexpression of chemerin increased levels of triglyceride (Fig. 2D) and cholesterol in the placenta (Fig. 2E).

**Overexpression of chemerin in trophoblast increased the expression of lipid-related proteins through CMKLR1/CCRL2 axis**

CMKLR1, GPR1 and CCRL2 are three chemerin recptors. The mRNA expression of CMKLR1 and CCRL2, but not GPR1 showed a significant increase in the placentas of mice with chemerin-overexpression (Fig. 3A-C). Notably, the expression of lipid-related genes *Pparg, Fabp4*, and *Srebp2* was also increased in the chemerin over-expressed placentas (Fig. 3D). The protein levels showed a similar increase (Fig. 3E and F). Furthermore, the protein levels of adiponectin, SREBP1 and the p-ERK/total ERK ratio were increased in the chemerin group's placenta (Fig. 3E and F). However, the protein levels of LDLR showed no significant change between the control and chemerin groups (Fig. 3E and F).

Accumulation of the lipid droplets in HTR-8/SVneo cells increased after the cells were induced a high level of chemerin (Fig. 4A). Chemerin overexpression increased the mRNA and protein levels of PPARg, FABP4, and SREBP2, as well as the protein level of ACC (Fig. 4B-F). Similar to the alterations observed in the chemerin-overexpressing placenta *in vivo*, adiponectin and SREBP1 protein levels and the p-ERK/total ERK ratio were increased (Fig. 4E and F). However, in the chemerin group, the protein levels of LDLR and its related protein SORT1 decreased (Fig. 4E and F).

**Overexpression of chemerin in trophoblast leads to increased production of lysophospholipids and phospholipids**

The results of phospholipidomics in HTR-8/SVneo cells revealed that the chemerin group produced significantly more lysophospholipids, specifically LPC, LPE, and LPG (Fig. 5A). Additionally, PA decreased while PC, PE, PG, PI, and PS were all increased in the chemerin group (Fig. 5A). More specifically, the levels of LPC species (16:1, 18:0 and 18:3), LPE species (16:0, 18:1, 18:2 and 18:3), and LPG 18:1 were increased in the chemerin group (Fig. 5B). Levels, PS species (34:1, 34:2, 36:1, 36:2, 36:3, 38:2, 38:3, 40:2, 40:3 and 40:4) were increased in the chemerin group (Fig. 5C). Levels of PC species (32:0, 34:2, 34:3, 36:1, 36:2, 36:3, 36:4, 36:5, 38:2, 38:3, 38:4, and 38:6) were increased in the chemerin group (Fig. 5D). Levels of PE species (32:1, 34:1, 34:2, 36:1, 36:2, 36:3, 36:4, 38:3, 38:5 and 38:6) were increased in the chemerin group (Fig. 5E). Levels of PI species (32:2, 32:3, 34:1, 34:2, 36:1, 36:2 and 36:3) were increased in the chemerin group (Fig. 5F). Levels of PA species (34:6 and 36:2) and PG species (32:1, 34:1, 34:2, 34:3, 34:4, 36:1, 36:2 and 36:3) were increased in the chemerin group (Fig. 5F).

## Discussion

The present finding disclosed that specific over-expression of chemerin in trophoblast-elevated the lipid levels in the mouse maternal serum and a placental lipid accumulation. Overexpression of chemerin also facilitated lipid accumulation in the human trophoblastic HTR-8/SVneo cells. Our previous research has reported that specific overexpression of chemerin in trophoblasts causes preeclampsia-like symptoms, and more chemerin is released in human preeclamptic placentas compared to the normal placentas [16]. The data suggest that elevated chemerin release in placenta might underlie the dyslipidemia occurring in preeclamptic patients, particularly in severe cases [4-8, 13-15].

Chemerin was initially discovered in adipocytes and liver, and associated with obesity, metabolic disorders, and cardiovascular disease [10-13, 15, 24, 25], with high levels in obese rodents and humans [24, 26, 27]. Chemerin promotes adipogenesis. Adipocyte differentiation and adipose tissue expansion were impaired when reducing chemerin level or blocking its receptor CMKLR1 [26, 28]. Elevated expression of CMKLR1 and CCRL2 in the specific chemerin-overexpressed trophoblasts suggests that these receptors are involved in the promotional effect of chemerin on lipid accumolation during pregnancy. The lipid elevation is associated with cholesterol rather than triglyceride, although the latter correlates positively with chemerin in metabolic disorders [29, 30].

It is well known that chemerin stimulates lipid accumulation in multiple cells, including 3T3-L1 cells and HepG2 cells [31, 32]. Investigations both *in vivo* and *in vitro* have shown that chemerin improved the lipid uptake and storage, evidenced by the enhanced production of PPARg [33], adiponectin [34], p-ERK1/2 [35-37], and FABP4 [38]. Chemerin increased cholesterol biosynthesis by upregulation of SREBP2 [39], and diminished LDL endocytosis via downregulating both the LDLR and SORT1 in HTR-8/SVneo trophoblast cells [40, 41], which subsequently reduced the LDL uptake and increased the cholesterol release. Moreover,

chemerin overexpression potentially improved the tricarboxylic acid (TCA) cycle and fatty acid synthesis by increasing ACC and SREBP1 [42, 43], which further enhanced the lipid deposition.

Pregnancy diseases, such pre-eclampsia, are coupled with lipid metabolic dysregulation, which is manifested by increase in maternal plasma lipid levels. The content of total phospholipid, individual phospholipid classes as well the cholesterol were increased in pre-eclamptic placental tissues [44, 45]. The maternal blood lipidome may be involved in the pathophysiology of severe preeclampsia and act as a biomarkers [8]. Oxidation products (e.g., oxLDL) derived from cholesterol and lysophospholipids contribute to the pathogenesis of preeclampsia and cause oxidative stress, eNOS dysfunction, endothelial dysfunction, and acute atherosclerosis [46]. Normal HDL has been described as an atheroprotective particle, but in patients with cardiovascular disease, HDL can be converted to a dysfunctional form and even exhibit proatherogenic effects [47]. Dysfunctional HDL is characterized by oxidized phospholipids and lysophospholipids, thereby losing its ability to promote cholesterol efflux and prevent LDL oxidation [48]. In this study, we found that chemerin overexpression increased the levels of phospholipids, lysophospholipids, and cholesterol in maternal blood and the placenta, while these materials accumulated in the labyrinth layer. The phospholipidomic findings demonstrated that high level of chemerin in human trophoblasts enhanced the contents of lysophospholipid molecular species of LPC 16:1 and18:2, and LPE18:2. Similar changes have been reported in blood of the preeclamptic women [8, 49]. Additionally, chemerin overexpression enhanced LPC 16:0, PE 34:2, and PE 34:3 levels, which also occur in preeclamptic placentas [50].

In preeclampsia condition, high level of chemerin is released from trophoblasts in the placenta, which increases the content of placental lipids (triglycerides, cholesterol, and phospholipids), lipid droplet accumulation, the TCA cycle and abnormal HDL through

CMKLR1/CCRL2 axis. Meanwhile, chemerin inhibits LDL uptake by reducing LDLR and SORT1 in trophoblasts, which leads to an increase in the release of lipids and the lipid-related protein (triglycerides, cholesterol, phospholipids, and chemerin) from the placenta to maternal circulation, as well as a lower LDL uptake from circulation to placenta, eventually resulting in dyslipidemia in the patient (Fig. 6).

**Comparisons with other studies and what does the current work add to the existing knowledge**

Many studies studied the changes of lipids in the circulation and placenta via comparision the healthy control with preeclampsia, but few of them focused on the mechanism underlying the dyslipidemia in preeclampsia. It is known that chemerin play important roles in lipid metabolism of adipocytes and hepatocytes. However, its effects on lipid accumulation in preeclampsic placenta has not been studied. Herein, the present study uses the preeclampsia-like mouse model induced by chemerin and chemerin-overexpressed human trophoblasts to validate its multiple roles in preeclampsia, which is helpful in suggesting chemerin as a potential target in clinical therapy.

**Study strength and limitations**

In our previous work, we developed a preeclampsia-like mouse model by specifically overexpressing chemerin in placenta. The present study dislosed a high level lipid in circulation and the local lipid accumulation in junctional zone and labyrinth in the placenta in this animal model, as well the alteration of phospholipds in the chemerin over-expressing human placental trphoblasts cells. The data give insight into pathogenesis of dyslipidemia during pregnancy which may be associated with lipid metabolism disfunction in placenta. However, it is difficult to mediate precisely the expression of chemerin via the lentiviral

system. Due to a lack of samples, phospholipid profile in palcenta was not analyzed. In addition, increasing HDL-C levels in the preeclampsia-like mice also need to be explored.

## Conclusions

In conclusion, this study suggests that chemerin disturbs trophoblast lipid metabolism by increasing lipid uptake, lipid droplet deposition, cholesterol biosynthesis, and by inhibiting LDL endocytosis and the TCA cycle, possibly via the CMKLR1/CCRL2 axis. A lipidome analysis in human placentas with high expression of chemerin may help to rich the roles of chemerin in pathogenesis of preeclampsia.

ORIGINALITY REPORT

| 8% | 5% | 5% | 1% |
| --- | --- | --- | --- |
| SIMILARITY INDEX | INTERNET SOURCES | PUBLICATIONS | STUDENT PAPERS |

PRIMARY SOURCES

| | | |
| --- | --- | --- |
| 1 | link.springer.com<br>Internet Source | 1% |
| 2 | www.researchgate.net<br>Internet Source | 1% |
| 3 | Sandeep Hans, Zeeshan Fatima, Saif Hameed. " Mass spectrometry based untargeted lipidomics reveals new compositional insights into membrane dynamics of under magnesium deprivation ", Journal of Applied Microbiology, 2021<br>Publication | 1% |
| 4 | www.science.gov<br>Internet Source | 1% |
| 5 | Hemin Jiang, Yuwei Liu, Yu Qian, Ziyang Shen, Yunqiang He, Rui Gao, Min Shen, Shu Chen, Qi Fu, Tao Yang. "CHL1 promotes insulin secretion and negatively regulates the proliferation of pancreatic β cells", Biochemical and Biophysical Research Communications, 2020<br>Publication | 1% |

| | | |
|---|---|---|
| 6 | Wei Li, Fengxiao Zhang, Chenhui Ju, Suying Lv, Kai Huang. "The role of CD27-CD70 signaling in myocardial infarction and cardiac remodeling", International Journal of Cardiology, 2018<br>Publication | <1% |
| 7 | docksci.com<br>Internet Source | <1% |
| 8 | repository.ajou.ac.kr<br>Internet Source | <1% |
| 9 | Stergios S. Bibis, Kelly Dahlstrom, Tongtong Zhu, Rachel Zufferey. "Characterization of Leishmania major phosphatidylethanolamine methyltransferases LmjPEM1 and LmjPEM2 and their inhibition by choline analogs", Molecular and Biochemical Parasitology, 2014<br>Publication | <1% |
| 10 | www.spandidos-publications.com<br>Internet Source | <1% |
| 11 | Submitted to Facultad Latinoamericana de Ciencias Sociales (FLACSO) - Sede Ecuador<br>Student Paper | <1% |
| 12 | Kim, S.J.. "Defective cholesterol traffic and neuronal differentiation in neural stem cells of Niemann-Pick type C disease improved by valproic acid, a histone deacetylase inhibitor", | <1% |

Biochemical and Biophysical Research Communications, 20070831
Publication

| | | |
|---|---|---|
| 13 | ash.silverchair-cdn.com<br>Internet Source | <1% |
| 14 | cellandbioscience.biomedcentral.com<br>Internet Source | <1% |
| 15 | mdpi.com<br>Internet Source | <1% |
| 16 | online.medunigraz.at<br>Internet Source | <1% |
| 17 | www.mdpi.com<br>Internet Source | <1% |
| 18 | www.frontiersin.org<br>Internet Source | <1% |
| 19 | Ruth Welti, Weiqi Li, Maoyin Li, Yongming Sang, Homigol Biesiada, Han-E Zhou, C. B. Rajashekar, Todd D. Williams, Xuemin Wang. "Profiling Membrane Lipids in Plant Stress Responses", Journal of Biological Chemistry, 2002<br>Publication | <1% |
| 20 | Wilson Rios-Munoz. "Fibroblast growth factor 2 applied to the optic nerve after axotomy increases Bcl-2 and decreases Bax in ganglion cells by activating the extracellular signal- | <1% |

regulated kinase signaling pathway", Journal of Neurochemistry, 6/2005
Publication

| | | | |
|---|---|---|---|
| Exclude quotes | On | Exclude matches | Off |
| Exclude bibliography | On | | |
